# Relationship between platelet activating factor acetylhydrolase activity and apolipoprotein B levels in patients with peanut allergy

**DOI:** 10.1186/1710-1492-10-20

**Published:** 2014-04-28

**Authors:** Boris Perelman, Areej Adil, Peter Vadas

**Affiliations:** 1 Division of Allergy and Clinical Immunology, Department of Medicine, St. Michael’s Hospital, University of Toronto, 30 Bond St., M5B 1 W8 Toronto, ON, Canada

**Keywords:** Anaphylaxis, Platelet-activating factor (PAF), PAF-acetylhydrolase (PAF-AH), Low density lipoprotein (LDL), Apolipoprotein B (apoB)

## Abstract

**Background:**

Platelet-activating factor (PAF) is a highly potent phospholipid mediator responsible for the life-threatening manifestations of anaphylaxis. PAF acetylhydrolase (PAF-AH) inactivates PAF and protects against severe anaphylaxis whereas deficiency of PAF-AH predisposes to severe or fatal anaphylaxis. Determinants of PAF-AH activity have not been studied in patients with peanut allergy.

**Objectives:**

To determine whether plasma PAF-AH activity in patients with peanut allergy is related to formation of circulating complexes with apolipoprotein B (apoB) the main surface protein on low density lipoprotein particles.

**Methods:**

Plasma PAF-AH activity and apoB concentrations were measured in 63 peanut allergic patients (35 boys, 28 girls, ages 2 – 19 years). ApoB concentration was measured immunoturbidimetrically using goat anti-human apoB. The correlation between PAF-AH activity and apoB concentration was determined.

**Results:**

A positive correlation was found between PAF-AH activity and apoB concentration (r^2^ = 0.59, P < 0.0001).

**Conclusion:**

In peanut allergic patients, PAF-AH activity strongly correlates with apoB concentration, suggesting the presence of circulating PAF-AH- lipoprotein complexes.

## Findings

Deficiency of platelet activating factor acetylhydrolase (PAF-AH) predisposes to severe or fatal anaphylaxis. PAF-AH correlates with apolipoprotein B in peanut allergic patients, suggesting that formation of PAF-AH-lipoprotein complexes is an important determinant of PAF-AH activity.

## Introduction

Platelet-activating factor (PAF) is one of the most potent lipid messengers involved in inflammatory events. PAF has been implicated in mediating the life-threatening manifestations of anaphylaxis including hypotension, increased vascular permeability, and severe bronchoconstriction [1,2]. The association of PAF with severe or fatal anaphylaxis has been observed in both animal models and in humans. PAF receptor antagonists protected against fatal anaphylaxis in experimental animals [3,4]. PAF receptor knockout mice were resistant to fatal anaphylaxis as compared to wild type mice [5]. In humans, PAF levels correlated more strongly with anaphylaxis severity than did histamine or tryptase [6,7]. In human vascular smooth muscle cells, epinephrine blocked PAF mediated signaling, likely by phosphorylation of the PAF receptor [8].

Serum PAF concentrations are rigorously controlled by tight regulation of biosynthesis and degradation. Cells synthesize and secrete PAF de novo only when stimulated. De-acetylation of PAF at the sn-2 position terminates the biologic activity of PAF, with formation of the inactive metabolite, lysoPAF [9]. The de-acetylation reaction is catalyzed by PAF acetylhydrolase (PAF-AH), a calcium-independent phospholipase A2 [10]. PAF-AH activity is an important determinant of the circulating half-life of PAF. The half-life of exogenous PAF is significantly longer in sera of PAF-AH deficient persons [6,11]. Conversely, increasing concentrations of PAF-AH correlate with more rapid inactivation of PAF [6]. Patients deficient in PAF-AH have been shown to be at increased risk of severe or fatal anaphylaxis, although the mechanisms regulating PAF-AH activity in patients at risk for fatal anaphylaxis have not yet been defined [6].

The circulating form of PAF-AH is also known as lipoprotein-associated phospholipase A_2_. In human plasma, 70% of PAF-AH circulates in fully active form as a complex with low density lipoprotein (LDL) and the remainder in high density lipoproteins (HDL) [12]. Plasma PAF-AH concentration directly correlates with LDL cholesterol concentrations in male subjects [13], such that changes in LDL concentration are reflected by corresponding changes in PAF-AH concentration. The catalytic activity of PAF-AH is regulated by its association with LDL, as lowering LDL in plasma increases the half-life of PAF [13]. The half-life of PAF is also prolonged in patients with abetalipoproteinemia, a condition characterized by deficiency of apolipoprotein-containing lipoproteins, including LDL [13]. Drugs that lower LDL levels lower PAF-AH activity as well. For example, rabbits treated with simvastatin for two months showed decreased PAF-AH activity as compared with control rabbits [14]. Although PAF-AH predominantly circulates in the blood associated with LDL in normolipidemic individuals, the relative proportion of PAF-AH associated with LDL and HDL can be affected by various factors [13]. The relationship between PAF-AH activity and LDL concentrations has not been studied in patients with a history of peanut allergy.

Apolipoprotein B (apoB) is complexed mainly with LDL and is a good surrogate measure of LDL concentration [15]. Generally, more than 90% of plasma apoB is on LDL particles (the remainder on LDL precursors). Each LDL precursor particle has exactly one molecule of apoB on its surface, which remains there during shrinkage to an LDL particle [16]. We undertook this study to investigate whether there is a correlation between PAF-AH activity and apolipoprotein B concentrations in patients with peanut allergy.

## Methods

### Study design

Apolipoprotein B concentrations and PAF-AH activity were measured in 63 children with peanut allergy (35 boys and 28 girls; mean age, 6.5 ± 3.9 years; range 2–19 years). These children had previous allergic reactions to peanut characterized only by urticaria and/or angioedema with positive skin tests to peanut protein of ≥ 8 mm wheal diameter and/or peanut-specific IgE ≥ 14 kU/L.

The demographics and diagnostic criteria for this patient cohort were reported previously [6]. Informed consent was obtained from all patients or their parents or guardians. This study was approved by the Research Ethics Board of St. Michael’s Hospital.

### Reagents

Radiolabelled PAF (1-O-hexadecyl-2-acetyl-H]-sn-glycero-3-phosphoholine, 499.5 Gbq/mmol) was purchased from NEN Life Science Products (Boston, MA). Unlabelled PAF and lyso-PAF were from BIOMOL (Brockville, Ontario). 1-palmitoyl-2-arachidonoyl-sn-glycero-3-phosphocholine, egg yolk phosphatidylcholine, pefabloc (4-2-[aminoethyl] benzenesulfonyl fluoride) and apolipoprotein-B kit were obtained from Sigma (Oakville, Ontario). Pre-coated TLC plates SILICA GEL 60 (layer thickness 0.25 mm, 20 × 20 cm) were from Merck, Germany. All other chemicals were from Sigma.

### Measurement of Apolipoprotein-B concentrations

Plasma PAF-AH activity has been shown to correlate strongly with the plasma concentration of low density lipoproteins (LDL) [10]. PAF-AH activity was measured as a function of apoB concentration. Human apoB concentration was measured immunoturbidimetrically using goat anti-human apoB (Sigma), which forms an insoluble complex resulting in turbidity of the assay mixture. Turbidity was measured spectrophotometrically at 340 nm. The concentration of apoB in the samples was determined from a calibration curve using multiple-level apoB calibrators (Sigma) at 5 different concentrations, ranging from 0–182 mg/dl.

### Measurement of PAF-AH activity

PAF-acetylhydrolase activity was measured according to the method of Miwa et al [2] as modified by Vadas et al [6].

## Results

### PAF-AH and apoB concentrations in patients with peanut allergy

**Figure 1:**
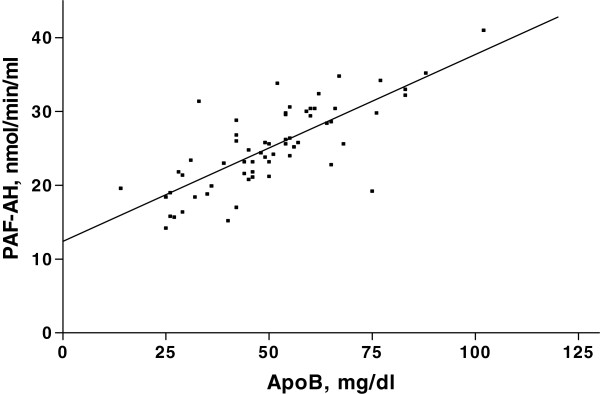
Scatterplot of PAF-AH activity and apolipoprotein B concentrations.

PAF-AH activity and apoB concentrations were measured in 63 peanut allergic patients. The relationship between PAF-AH activity, peanut-specific IgE levels and apoB concentrations is shown in Table 1. PAF-AH activities were plotted as a function of apoB concentrations. Plasma PAF-AH activity was strongly correlated with the plasma concentrations of apoB (r^2^ = 0.59, P < 0.0001) (Figure 1).

**Table 1:** PAF-AH, peanut-specific IgE and ApoB concentrations in patients with peanut allergy

	**PAF-AH nmol/min/ml**	**Peanut-specific IgE KU/L**	**ApoB mg/dl**
Mean	25.18	110.5	50.51
Standard deviation	5.67	270.60	17.17
Standard error	0.7150	34.09	2.163
Lower 95% CI of mean	23.75	42.39	46.18
Upper 95% CI of mean	26.61	178.70	54.83

## Discussion

Recently, we reported that in patients with acute allergic reactions, blood PAF levels correlated with severity of anaphylaxis. Patients with the highest levels of circulating PAF had more severe reactions and, conversely, those with lowest PAF levels had least severe reactions [6]. PAF-AH activity correlated inversely with PAF levels and patients with the lowest levels of PAF-AH were at highest risk of severe or fatal anaphylaxis [6]. The odds ratio for patients with severe anaphylaxis vs patients with mild – moderate reactions was 27.0 (95% CI, 4.2-175.5; 2 tailed P = 0.00005 for uncorrected Chi-square) [17]. An odds ratio of 27.0 suggests that patients with the lowest levels of PAF-AH activity were 27.0 times more at risk of severe or fatal anaphylaxis, as compared to patients with normal levels of PAF-AH activity. These and other studies [18] suggest that PAF-AH deficiency predisposes to severe anaphylaxis.

PAF is a potent pro-inflammatory phospholipid messenger which acts via a trans-membrane G-protein coupled PAF receptor [11]. The production of PAF is tightly regulated by both synthetic and degradative processes. Whereas synthesis of PAF in activated cells involves at least 2 enzymes, an arachidonate-specific phospholipase A_2_ (PLA_2_) and acetyl-CoA-lyso-PAF acetyltransferase, the degradation of PAF to the biologically inactive form, lyso-PAF, is accomplished by the enzyme PAF-AH. The most likely function of PAF-AH may be that of a safety mechanism to limit the levels of PAF [19].

In contrast to proinflammatory phospholipases [20,21], PAF-AH is an anti-inflammatory phospholipase that occurs as a plasma isoform complexed mainly with LDL. In a cohort of 240 normolipidemic individuals, plasma PAF-AH activity was strongly correlated with the plasma concentration of LDL [22]. Among normolipidemic individuals, treatment with lovastatin and fenofibrates resulted in proportionate decreases in plasma PAF-AH activity and LDL concentrations [22,23]. Removal of LDL from the circulation may determine the clearance rate of PAF-AH, modulating the activity of PAF-AH in blood, and thereby influencing the level of PAF [22].

In this study, our aim was to investigate whether PAF-AH activity correlates with apoB, the main surface protein on LDL particles, in patients with peanut allergy. We did find a linear relationship between serum PAF-AH and apoB concentrations throughout the range of PAF-AH activities, in a pediatric patient population. Complex formation with lipoproteins, especially LDL, may, therefore, be an important determinant of PAF-AH activity. Dietary or pharmacologic strategies to lower LDL may have the unintended consequence of altering PAF-AH activity. In particular, lowering LDL levels pharmacologically in patients at risk for anaphylaxis may lower PAF-AH activity in these patients as well, inadvertently leading to an increased risk of severe or fatal anaphylaxis. It remains to be seen if the results reported for a pediatric age group can be extended to adult patients who are most likely to be using lipid lowering drugs. A study of the relationship between use of lipid lowering drugs to severity of anaphylaxis is currently in progress.

## Abbreviations

apoB: Apolipoprotein B; LDL: Low density lipoproteins; PAF-AH: Platelet activating factor acetylhydrolase; PAF: Platelet activating factor; PLA2: Phospholipase A_2_.

## Competing interests

Peter Vadas has been granted US Patent 8562982: Use of platelet activating factor acetylhydrolase as a biomarker for anaphylaxis.

## Authors’ contributions

BP carried out the immunoturbidimetric studies. AA participated in drafting the manuscript. PV designed the studies, carried out the statistical analysis and drafted the manuscript. All authors read and approved the final manuscript.
